# Ezrin regulates synovial angiogenesis in rheumatoid arthritis through YAP and Akt signalling

**DOI:** 10.1111/jcmm.16877

**Published:** 2021-08-29

**Authors:** Qiyue Chen, Kai Fan, Xi Chen, Xiaobo Xie, Li Huang, Guangbao Song, Weizhong Qi

**Affiliations:** ^1^ Department of Special Clinics Stomatological Hospital Southern Medical University Guangzhou Guangdong China; ^2^ Department of Dermatology Nanfang Hospital Southern Medical University Guangzhou Guangdong China; ^3^ Department of Joint and Orthopedics Zhujiang Hospital Southern Medical University Guangzhou Guangdong China

**Keywords:** angiogenesis, Ezrin, Hippo‐YAP, PI3K/Akt, rheumatoid arthritis

## Abstract

This study aimed to investigate the role and regulatory mechanisms of Ezrin in synovial vessels in rheumatoid arthritis (RA). Synovial tissues were obtained from people with osteoarthritis people and patients with RA patients. We also used an antigen‐induced arthritis (AIA) mice model by using Freund's adjuvant injections. Ezrin expression was analysed by immunofluorescence and immunohistochemical staining in synovial vessels of patients with RA and AIA mice. We investigated the role of Ezrin on vascular endothelial cells and its regulatory mechanism *in vivo* and *in vitro* by adenoviral transfection technology. Our results suggest a role for the Ezrin protein in proliferation, migration and angiogenesis of vascular endothelial cells in RA. We also demonstrate that Ezrin plays an important role in vascular endothelial cell migration and tube formation through regulation of the Hippo‐yes‐associated protein 1 (YAP) pathway. YAP, as a key protein, can further regulate the activity of PI3K/Akt signalling pathway in vascular endothelial cells. In AIA mice experiments, we observed that the inhibition of Ezrin or of its downstream YAP pathway can affect synovial angiogenesis and may lead to progression of RA. In conclusion, Ezrin plays an important role in angiogenesis in the RA synovium by regulating YAP nuclear translocation and interacting with the PI3K/Akt signalling pathway.

## 1 INTRODUCTION

Rheumatoid arthritis (RA) is a complex chronic autoimmune disease that involves inflammation of multiple joints in the body. RA is characterized by persistent and progressive synovitis and joint erosion, and can progress to lead to joint deformity and functional loss.^1^ Histopathological features of RA include synovial hyperplasia, pannus formation and destruction of the cartilage and bone. Pannus formation is the main manifestation during the pathological process of RA. Pannus is a type of extra growth mainly composed of new microvessels, hypertrophic synovial cells, inflammatory cells and organized cellulose.^2^ Pannus can release immunoglobulin and rheumatoid factors, and participate in synovial inflammation and tissue proliferation, leading to the destruction of cartilage, bone, tendon, ligament and blood vessels.^3^, ^4^

Ezrin is a member of ERM (i.e. Ezrin, Radixin and Moesin) protein family. It is an important junction protein between the cell membrane and actin cytoskeleton. It plays an important role in cell morphology maintenance, cell movement, adhesion, signal transduction, apoptosis regulation, and metastasis promotion and prognosis of malignant tumours.^5^, ^6^ According to recent literature, Ezrin has been implicated several endothelial cell‐related processes, such as endothelial cell proliferation and neovascularization,^7^ regulation of the water balance^8^ and maintenance of the endothelial cell pore size.^9^

Angiogenesis is a physiological complex process through which new microvessels form from the pre‐existing vascular system and involves endothelial cell activation, basement membrane rupture and migration, endothelial cell expansion, lumen formation and, finally, the formation of new blood vessels. Prior research literature has shown that the Hippo‐YAP signalling pathway plays a key role in angiogenesis, by inducing endothelial cell proliferation and angiogenesis, and promoting vascular development. However, to the best of our knowledge, no robust data have shown whether the Hippo‐YAP pathway regulates RA synovial angiogenesis.

In this study, we aimed to investigate the expression and role of Ezrin and YAP in vascular cells of synovial tissues of patients with RA and of antigen‐induced arthritis (AIA) mice. We showed that Ezrin regulates the formation of RA synovial blood vessels through the Hippo‐YAP pathway. In addition, in vitro experiments revealed that Ezrin can regulate vascular cell proliferation by affecting the nuclear translocation of YAP. On the other hand, YAP is a regulator of the PI3K/Akt signalling pathway, which in turn further regulates the migration, proliferation and tube formation of vascular cells. Finally, in vivo experiments confirmed that the inhibition of Ezrin or the Hippo‐YAP pathway can facilitate the control of synovial angiogenesis.

## 2 MATERIALS AND METHODS

### 2.1 Tissue Specimens

We included tissue specimens derived from ten individuals. Specifically, we included knee synovial specimens of patients with RA (*n* = 5; age =67.4 ± 5.3 years; one male and four female), and knee joint synovial specimens from patients with osteoarthritis (*n* = 5; age =69.2 ± 7.1 years; two male and three female) as controls. All synovial tissue samples were obtained from patients admitted to Zhujiang Hospital of Southern Medical University for total knee replacement. The diagnosis of rheumatoid joints complied with the revised standards of the American College of Rheumatology in 1987.^10^ All subjects provided informed consent. The experimental protocol was approved in advance by the Ethics Committee of Zhujiang Hospital of Southern Medical University, China.

### 2.2 Cell culture

Human Umbilical Vein Endothelial Cells (HUVECs) were purchased from Cell Applications (www.cellapplications.com/human‐umbilical‐vein‐endothelial‐cells‐HUVECs) and maintained in DMEM (Gibco, USA) with 10% FBS (Gibco), 100 U/ml penicillin and 100 mg/ml streptomycin sulphate (Life Technologies, Inc., Grand Island, NY, USA), at 37℃ with 5% CO_2_. Cells were treated with different conditions for 24 hr, and protein and RNA extraction were carried out.

### 2.3 Animals

Female C57/BL6 mice (6–7 weeks old, ~20 g body weight) were maintained on a standard chow pellet diet with had free access to water, on a 12 h light/dark cycle. Groups of five mice were used for each treatment to a total number of 40 animals. Animals were monitored daily for their health status. Experimental protocols were approved by the Committee of the Southern Medical University (Guangzhou, China), in accordance with the Institutional Animal Care and Use Committee guidelines. Animal experiments were carried out in accordance with the Declaration of Helsinki.

### 2.4 Adjuvant‐induced arthritis model and treatment

Female C57/BL6 mice (6–7 weeks old, 20 g body weight) were obtained from The Guangdong Laboratory Animal Center, Guangzhou, China. To induce RA in C57/BL6 mice, mice were immunized on day 0 with 200 μg methylated BSA (mBSA) (CFA, Sigma‐Aldrich) emulsified in 0.2 ml of Complete Freund's adjuvant heat‐killed Mycobacterium tuberculosis suspended in paraffin oil and mannide monooleate 1 mg/ml), which was injected subcutaneously into flank skin. A booster subcutaneous injection with 10 μg mBSA emulsified in 10 μg Freund's incomplete adjuvant (CFA, Sigma‐Aldrich) was given into the knee joint at day 7. Treatments were started on day 8. Verteporfin (VP) was administered intravenously at a dose of 1 mg/kg. The control group was injected with the same amount of normal saline at the same time. Intraarticular injection of adenovirus (5 μl) was performed once a week. Experimental protocols were approved by the Southern Medical University Committee in accordance with the Institutional Animal Care and Use Committee guidelines. The present study was carried out in accordance with the Declaration of Helsinki.

### 2.5 Assessment of arthritis severity

Histologic sections were scored based on a scale of 0–3 for each of the following five parameters: synovitis (defined as hypercellularity of the synovium), joint space exudate (defined as leukocytes in the joint space), soft tissue inflammation (defined as leukocyte infiltration of the infrapatellar fat pads, joint capsule and the area adjacent to the periosteal sheath), cartilage degradation (defined by loss of haematoxylin and eosin staining; 0 = full‐stained cartilage, 3 = fully unstained cartilage) and bone damage (defined according to the extent and depth of subchondral bone damage). The total histologic score was the sum of the scored of the individual arthritis features (up to a maximum of 15).^11^

### 2.6 Immunohistochemistry

Synovial tissue specimens from patients with RA were fixed in paraformaldehyde (PFA) and embedded in paraffin. Specimens were embedded in paraffin and 5 μm serial sections were performed for immunohistochemical staining based on immunoperoxidase technique with diaminobenzidine as the chromogen. Following deparaffinization, microwave antigen retrieval was performed by incubating sections in 10 mM EDTA pH 7.5 at 93℃ for 10 minutes, after which sections were allowed to cool for at least 20 min. Sections were then washed in Tris‐buffered saline (TBS) and incubated in serum block (10% FCS and 10% serum diluted in TBS) for 60 min. Sections were incubated with the primary antibody diluted in PBS (1:100) overnight at 4℃ in a humidified chamber. Sections were incubated with the secondary antibody at room temperature for 1 h. Finally, sections were counterstained with haematoxylin and eosin and mounted.

### 2.7 Immunofluorescence

For immunofluorescence analysis, PFA‐fixed plated cells were incubated with blocking solution (10% normal goat serum, 0.1% Triton ×100 in PBS), for 1 h at room temperature. Samples were then incubated with the primary antibody overnight at 4℃. For quantification of Ezrin, Ki67, YAP and DAPI colocalization, nuclear ROIs were segmented and the ratio of CD31‐positive area to the total area was quantified using Image J analysis software.

### 2.8 MTT test

To determine the effects of VP on cell proliferation, the 3‐(4,5‐dimethylthiazol‐2‐yl)‐2,5‐diphenyltetrazolium bromide (MTT) colorimetric assay was carried out. For the MTT assay, in brief, 1 × 10^4^ HUVECs/well were plated in 96‐well plates with 100 μl of culture medium overnight. 24 h later, the medium was replaced with fresh medium containing different concentrations of drugs. 20 μl (0.5 mg/ml) of MTT reagent was added to the cells at different time points and incubated for 2 h at 37℃. The medium was then removed, and 150 μl of DMSO was added to dissolve the formazan crystals formed. A BioTek plate reader was used to read absorbance at 570 nm.

### 2.9 Cell migration

Confluent cell monolayers were scraped with a pipette tip to generate a wound. Cell debris was removed by washing with PBS several times. Cell monolayers were photographed with a digital camera straight after the scratch would and 12 h after, and the degree of wound closure was determined with the Image J analysis software.

### 2.10 Tube formation

We assessed the drug effects on the ability of HUVECs to reorganize and differentiate into capillary‐like networks using an *in vitro* Matrigel morphogenesis assay. HUVECs (1.5 × 10^5^ cells/ml) were seeded in 48‐well plates pre‐coated with Matrigel (50 μl/well). Matrigel was then polymerized for 40 min at 37℃. After cell incubation for 6 h in serum‐containing media, images of tube morphology were captured using a microscope, and the extent of tube formation was quantified by counting the number of meshes with the Image J analysis software.

### 2.11 Western blot analysis

HUVECs were grown in 100‐mm dishes. HUVECs were trypsinized, pelleted at 300 g and then lysed with 150 μl RIPA lysis buffer and 1mM phenylmethylsulfonyl fluoride. Samples comprising equal amounts of total protein were analysed by 7.5% and/or 12% SDS‐PAGE and then transferred to a polyvinylidene difluoride (PVDF) membrane (Millipore). Following electrophoretic transfer, PVDF membranes were blocked with Protein Free Rapid Blocking Buffer (Beyotime, China). Blots were developed with an enhanced chemiluminescence reagent according to the manufacturer's protocol (Beyotime, China).

### 2.12 Adenovirus vector and transfection

Adenoviruses expressing or silencing Ezrin, YAP were purchased from Hanbio Technology Ltd (Shanghai, China). To achieve RNAi‐mediated depletion of Ezrin and YAP, cells were transfected with siRNA oligos targeting Ezrin and YAP (Si‐) or negative controls (Si‐Vector‐). A vector carrying the adenovirus (Ad)‐Ezrin or (Ad)‐YAP plasmid (oeEzrin, oeYAP) and negative control (NC) GFP vector carrying an empty plasmid were generated by Hanbio Technology Ltd (Shanghai, China).

HUVECs were infected with recombinant adenovirus expressing human Ezrin or YAP according to manufacturer's instructions. The adenoviruses titres used in this study were 1.2 × 10^8^ PFU/ml, and the multiplicity of infection was 20. Following adenoviral transfection, cells were cultured in DMEM medium for 48 h.

### 2.13 Quantitative real‐time PCR

Cultured cells were harvested using TRIzol reagent (Sigma, USA), and the mRNA was extracted from the TRIzol reagent using an RNA extraction kit (Invitrogen, USA) according to the manufacturer's instructions. We used reverse transcription reagents and real‐time PCR mix (Vazyme Biotech, China) on a LightCycler® 96 (Roche Life Sciences). The expression levels of all genes (Table 1) were determined relative to *glyceraldehyde*‐*3*‐*phosphate dehydrogenase* (*GAPDH*) gene expression. qPCRs were performed in triplicate from two independent experiments.

**TABLE 1:** Primers used for the qPCR analyses of mRNA expression

Gene	Primer forward	Primer reverse
*CTGF*	CCAAGGACCAAACCGTGGT	TACTCCACAGAATTT AGCTCG
*bFGF*	AAGAGCGACCCTCACATCA	TCGTTTCAGTGCCACATACC
*Cyr61*	CGAGGTGGAGTTGACGAGAA	GCACTCAGGGTTGTCATTGGT
*C‐myc*	GGCTCCTGGCAAAAGGTCA	CTGCGTAGTTGTGCTGATGT
*GAPDH*	TCA ACGGCACAGTCAAGG	ACTCCACGACATACTCAG C

### 2.14 Antibodies

The following antibodies were used: Ezrin (1:1000 for Western blot, CST, USA, 3145), Ki67 (1:100 for IF, Abcam, USA, ab15580), CD31 (1:200 for IHC, Abcam, USA, ab24590), YAP (1:200 for IHC/IF,1:1000 for Western blot, CST, USA, 14074T), PTEN (1:1000 for Western blot, CST, USA, 9188T), p‐S6 (1:1000 for Western blot, CST, USA, 4858T), S6 (1:1000 for Western blot, CST, USA, 2317T), Phospho‐Akt (1:1000 for Western blot, CST, USA, 4060T), Akt (1:1000 for Western blot, CST, USA, 9272T), CTGF(1:200 for IHC, CST, USA,86641S), Alexa 594 or 488 dye‐labelled secondary antibodies (Jackson ImmunoResearch 383 Laboratories, Inc.) and *GAPDH* (1:1000 for Western blot).

### 2.15 Statistical analysis

All statistics were performed using GraphPad Prism (version 5.00 for Mac, GraphPad Software, San Diego, CA, USA). Data were tested for normality using a D’Agostino and Pearson omnibus normality test and subsequently assessed for homogeneity of variance. Data that passed both tests were further analysed by a two‐tailed unpaired Student's t test for comparison of *n* = 2 groups. Comparisons of *n* > 2 groups were performed using a one‐way ANOVA and Holm‐Sidak's multiple comparisons test. For all statistical tests, *p*‐values <0.05 were considered statistically significant; *p*‐values are reported within the figure legends.

## 3 RESULTS

### 3.1 Ezrin expression and angiogenesis were increased in the synovial tissue patients of rheumatoid arthritis

**FIGURE 1:**
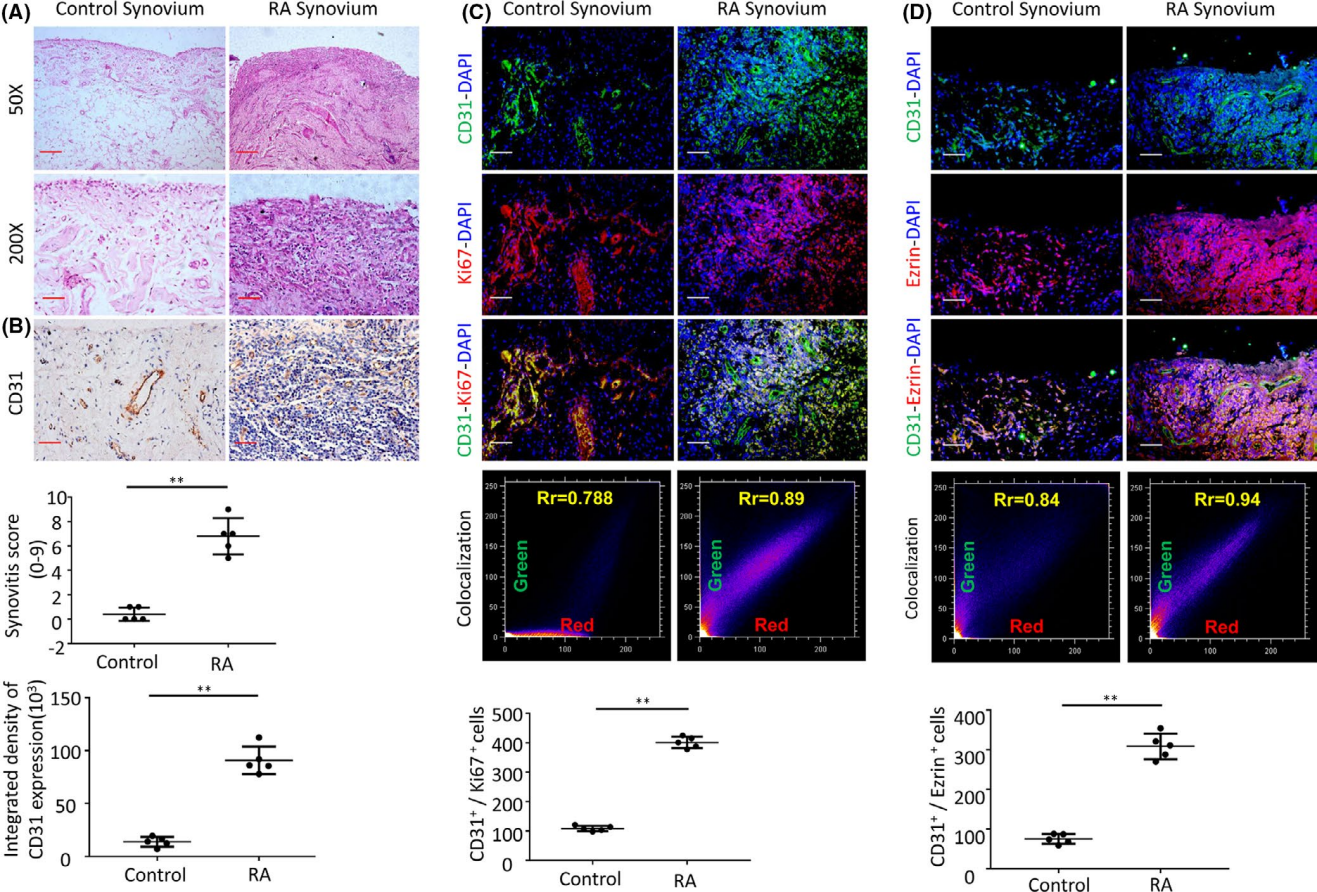
(A) Representative images of haematoxylin and eosin staining in RA human synovial tissues (Scale bar, 50 μm or 100 μm). (B) CD31 detection in the IHC analysis and positive area of the knee joints from human synovial tissues. (C) Representative immunofluorescence staining images and CD31&Ki67 colocalization, positive cells of CD31 and Ki67 in synovial tissues of RA patients (scale bars, 50 μm). (D) Representative immunofluorescence staining images and CD31&Ezrin colocalization, positive cells of CD31 and Ezrin in synovial tissues of RA patients (scale bars, 50 μm). *n* = 5, *p < 0.05, **p < 0.01

To investigate the synovial vessel alterations in RA, we first studied the angiogenesis of synovial vessels synovial tissues derived from patients with RA. Similar to previous studies, excessive synovial hyperplasia and abundant inflammatory cell infiltration were observed in haematoxylin and eosin‐stained synovial tissue from patients with RA. Moreover, enhanced angiogenesis was also observed in the synovial tissue, as assessed by immunohistochemical CD31^+^ staining and quantification of CD31 positivity (Figure 1A and 1B). There were significantly more CD31^+^ cells in the synovial tissues from the RA group than in the control group (13.3 ± 4.66 cells/mm^3^ vs. 84.82 ± 20.48 cells/mm^3^, *p* <0.01). Immunofluorescence staining showed more CD31 and Ki67 double staining positive cells in synovial tissues of patients with RA, respectively (108.6 ± 9.07 cells/mm^3^ and 401.4 ± 19.71 cells/mm^3^, *p* <0.01) (Figure 1C). It is believed that there are new blood vessels with strong proliferation ability. These results that the number and proliferation of synovial vascular endothelial cells in RA patients are increased. In addition, immunohistochemical staining demonstrated that Ezrin protein expression in the vascular endothelial cells of synovial tissues from patients with RA is increased compared to the control group (75.2 ± 12.44 cells/mm^3^ vs. 308.8 ± 32.15 cells/mm^3^, *p* <0.01) (Figure 1D). Ezrin and CD31 double staining confirmed that Ezrin was primarily expressed in the vascular endothelial cells of patients from both groups.

### 3.2 Ezrin expression and angiogenesis were increased in the synovial tissue of AIA‐treated mice

**FIGURE 2:**
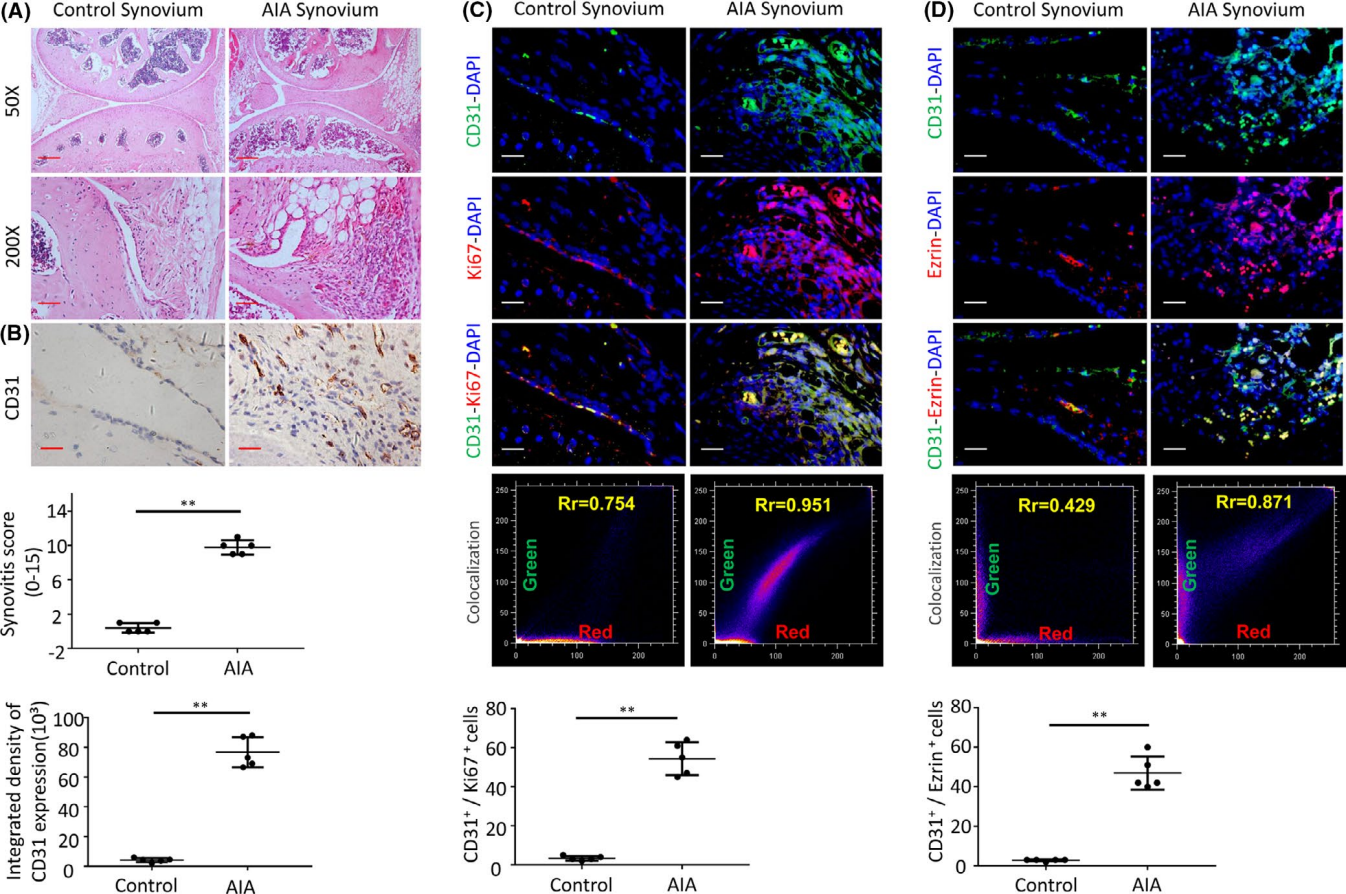
(A) Representative images of haematoxylin and eosin staining in AIA‐treated mice (Scale bar, 50 μm or 25 μm). (B) CD31 detection in the IHC analysis and positive area of the knee joints from AIA‐treated mice. (C) Representative immunofluorescence staining images and CD31&Ki67 colocalization, positive cells of CD31 and Ki67 in synovial tissues of AIA‐treated mice (scale bars, 25 μm). (D) Representative immunofluorescence staining images and CD31&Ezrin colocalization, positive cells of CD31 and Ezrin in synovial tissues of AIA‐treated mice (scale bars, 25 μm). *n* = 5, *p < 0.05, **p < 0.01

To confirm our preliminary results obtained from synovial tissue of RA patients, we performed animal experiments. After inducing arthritis in mice by administration of complete/incomplete Freund's adjuvant, we observed different degrees of proliferation, infiltration of numerous inflammatory cells in the synovial membrane and increased formation of the synovial blood vessels the synovial tissues (Figure 2A). Immunohistochemical staining demonstrated that the number of CD31^+^ cells in the synovial tissues of AIA mice was increased compared to control mice, which is due to increased proliferation of vascular endothelial cells. There were significantly more CD31^+^ cells in synovial tissues in the AIA group than in the control group (3.7 ± 1.12 cells/mm^3^ vs. 74.5 ± 9.03 cells/mm^3^, *p* <0.01) (Figure 2B). Immunofluorescence staining indicated more proliferating CD31 and Ki67 double staining positive cells in the synovial tissues of AIA mice compared to control mice (3.4 ±1.14 cells/mm^3^ vs. 54.5 ± 8.36 cells/mm^3^, *p* <0.01) (Figure 2C). Ezrin expression was detected in synovial vessels of AIA mice and was significantly increased in the synovial vascular endothelial cells of AIA mice in comparison with control mice (2.8 ± 0.45 vs. 47 ± 8.43, *p* <0.01) (Figure 2D).

### 3.3 Blocking Ezrin inhibits angiogenesis and delays arthritis progression in AIA mice

**FIGURE 3:**
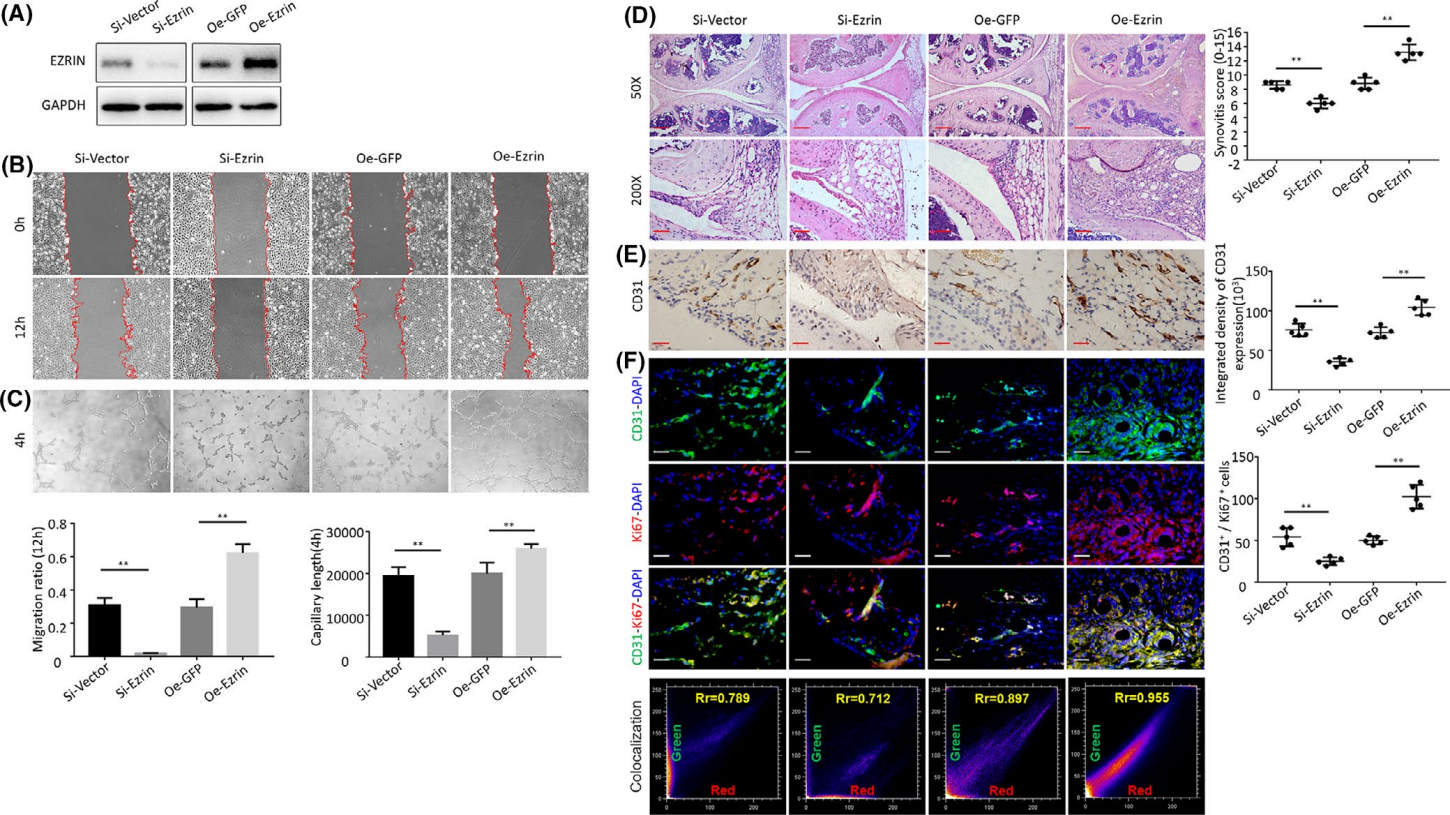
(A) The Western blot was used to measure Ezrin protein in HUVECs following transfection and silencing of Ezrin. (B) Tube formation assay of HUVECs following transfection and silencing of Ezrin. (C) Representative images of Wound‐healing assay of HUVECs following transfection and silencing of Ezrin. (D) Representative images of haematoxylin and eosin staining in AIA‐treated mice after intraarticular injection of adenovirus (Scale bar, 50 μm or 100 μm). (E) CD31 detection in the IHC analysis and positive area of the knee joints from treated AIA mice after intraarticular injection of adenovirus (Scale bar, 25 μm). (F) Representative immunofluorescence staining images and CD31&Ki67 colocalization, positive cells of CD31 and Ki67 in synovial tissues of AIA‐treated mice after intraarticular injection of adenovirus (scale bars, 25 μm). *n * = 5, *p < 0.05, **p < 0.01

To explore the potential role of Ezrin in arthritis, Ezrin expression was depleted by siRNA‐mediated knockdown and increased through oeRNA‐mediated overexpression in HUVECs (Figure 3A). To identify the potential function of Ezrin in HUVECs migration, we assessed wound closure in the cultured cells. The percentage of wound closure was significantly greater in the Ad‐Ezrin‐transfected cells than in the Ad‐GFP‐transfected cells, whilst the wound closure in Ezrin siRNA‐transfected cells was significantly lower than that in Si‐vector‐transfected cells (Figure 3B). These results suggest that the capacity of HUVECs migration in vitro may be influenced by Ezrin siRNA‐mediated knockdown and increased by RNA‐mediated overexpression.

To investigate the role of Ezrin in the regulation of HUVECs proliferation, formation of tubular networks was assessed. Our results reveal that organization into cords was reduced by Ezrin siRNA‐mediated knockdown and increased by oeRNA‐mediated overexpression (Figure 3C). To explore the effect of Ezrin expression on AIA joint synovitis and synovial vessels, we injected Ad‐Ezrin and control vector into the articular cavity of AIA mice for 6 weeks, once a week. Histological staining showed partial remission of synovitis in AIA mice in the Ezrin knockdown group (Figure 3D). In addition, CD31 immunostaining showed a decrease CD31 positivity and a decrease in blood vessels in the synovial tissue in the Ezrin knockdown group (Figure 3E). Immunofluorescence staining showed that the proliferation of vascular endothelial cells (CD31+ cells) and the number of CD31 and Ki67 double‐positive cells decreased in the Ezrin knockdown group (Figure 3F). In contrast, in the Ezrin overexpression group, synovitis was aggravated and signs of synovial vascularization were evident.

### 3.4 Ezrin regulates YAP expression

To explore the signalling pathways downstream of Ezrin, we hypothesized that Ezrin may affect the proliferation and differentiation of vascular endothelial cells. Previous studies found a role for the Hippo‐YAP pathway is proliferation and differentiation in a variety of diseases. However, it is unknown whether the Hippo‐YAP pathway is activated in the synovial tissue of RA. Therefore, we set out to explore how manipulations of Ezrin impacted the Hippo‐YAP pathway.

**FIGURE 4:**
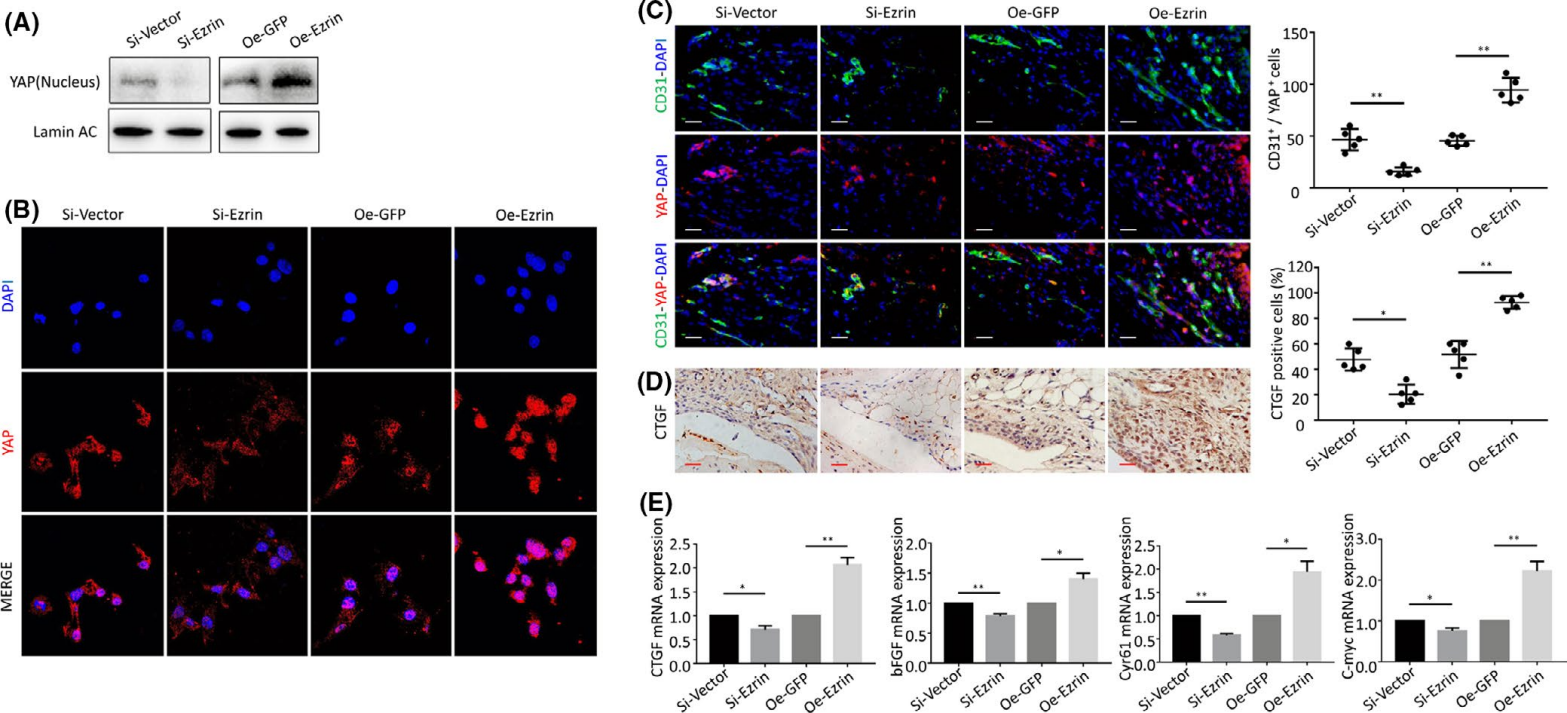
(A) The Western blot was used to measure YAP protein expression in HUVECs n nucleus and cytoplasm following transfection and silencing of Ezrin. (B) Representative images of nuclear translocation of YAP. (C) Representative immunofluorescence staining images and positive cells of CD31 and YAP in synovial tissues of AIA‐treated mice after intraarticular injection of adenovirus. (D) CTGF protein levels in the IHC analysis and positive cells of the knee joints from inhibition and overexpression of Ezrin AIA mice (Scale bar, 25 μm). (E) The qPCR analysis of CTGF, bFGF, Cyr61 and C‐myc mRNA expression. *n *= 5, *p < 0.05, **p < 0.01

Western blotting on both the nuclear and cytosolic fractions indicated that nuclear YAP was significantly increased upon Ezrin overexpression and reduced upon Ezrin knockdown (Figure 4A). Immunostaining indicated that YAP is primarily localized in the nuclei cells. Ezrin siRNA cells significantly impaired YAP nuclear translocation, whereas Ezrin overexpression significantly promoted YAP nuclear translocation (Figure 4B). In addition, immunohistochemical staining showed a significant increase in the number of CD31^+^ cells in synovial vessels of AIA mice injected with Ezrin overexpression adenovirus. YAP and CD31 double staining indicated that YAP was mainly expressed in the vascular endothelial cells of AIA mice. In contrast, Ezrin was knocked down. YAP expression in synovial vascular endothelial cells decreased significantly in AIA mice (Figure 4C).

The activation or knockdown of YAP can lead to gene changes such as proliferation and migration. Immunohistochemical detection of CTGF, a marker of activation of proliferation, migration and angiogenesis showed that Ezrin silencing decreased CTGF expression. Knockdown of Ezrin led to significantly less CTGF positive cells in synoviocytes and decreased angiogenesis. In contrast, Ezrin overexpression showed a significantly increased number of CTGF positive cells in the synovial vessels of AIA mice (Figure 4D). We then investigated the effect of Ezrin in the levels of YAP downstream genes involved in regulating cell growth and proliferation, such as CTGF, bFGF, Cyr61 and C‐myc in different groups of HUVECs by qPCR (Figure 4E).

### 3.5 Blocking the Hippo‐YAP pathway inhibits angiogenesis and delays progression of arthritis in AIA mice

**FIGURE 5:**
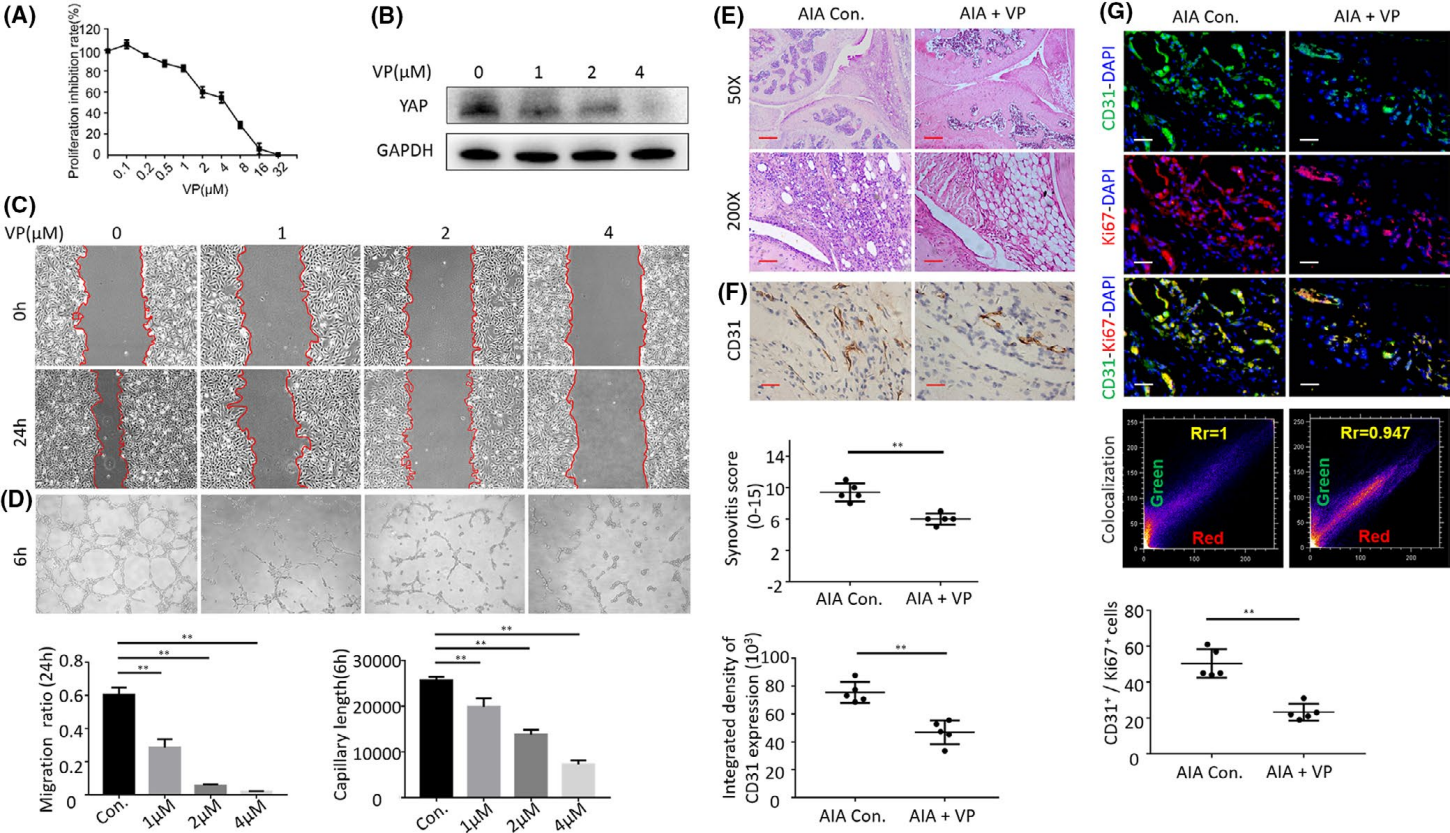
(A) Inhibition of proliferation of verteporfin was measured with MTT assay. (B) The Western blot results showed that the verteporfin treatment inhibited the protein expression of YAP in HUVECs. (C) Representative images of wound‐healing assay. (D) Tube formation assay of HUVECs treated with verteporfin. (E) Representative images of haematoxylin and eosin staining in verteporfin treatment on AIA mice (Scale bar, 50 μm or 100 μm). (F) CD31 detection in the IHC analysis and positive area of the knee joints from treated AIA mice (Scale bar, 25 μm). (G) Representative immunofluorescence staining images and CD31&Ki67 colocalization, positive cells of CD31 and Ki67 in synovial tissues of treated AIA mice (scale bars, 50 μm and 25 μm). *n* = 5, *p < 0.05, **p < 0.01

In order to investigate the effect of activation of the Hippo‐YAP pathway on the synovitis and synovial vessels of AIA mice, we selected VP, an inhibitor of Hippo‐YAP. First, CCK8 was used to detect the effect of VP in inhibiting cell proliferation. A proliferation inhibition rate >50% was accordingly selected, and 1, 2, and 4 μM were selected as the experimental concentrations for subsequent experiments (Figure 5A). Western blotting revealed that VP inhibited YAP expression (Figure 5B), tube formation (Figure 5C), and migration (Figure 5D) of the vascular endothelial cells in a concentration‐dependent manner. We then administered VP via intraperitoneal injection in AIA mice during 6 weeks and inspected the synovial tissues of the knee joint. Haematoxylin and eosin staining revealed that the synovial blood vessels decreased, the proliferation of the synovial cells decreased, the synovitis score decreased, and the degree of synovitis was partially relieved in AIA mice compared to the control group (Figure 5E). Immunohistochemical staining suggested that the expression of CD31 decreased in the synovial tissues of AIA upon VP treatment. There were significantly less CD31^+^ cells in the synovial tissues in VP group than in the AIA group (73.2 ± 7.54 cells/mm^3^ vs. 42.9 ± 16.92 cells/mm^3^, *p* <0.01) (Figure 5F). In addition, the proliferation of CD31^+^ vascular endothelial cells and the number of CD31 and Ki67 double‐positive cells decreased (Figure 5G) in AIA mice compared to the control group (50.4 ± 7.99 cells/mm^3^ and 23.2 ± 4.60 cells/mm^3^, *p* <0.01).

### 3.6 Hippo‐YAP pathway and PI3K/Akt pathway interaction in vascular endothelial cells

**FIGURE 6:**
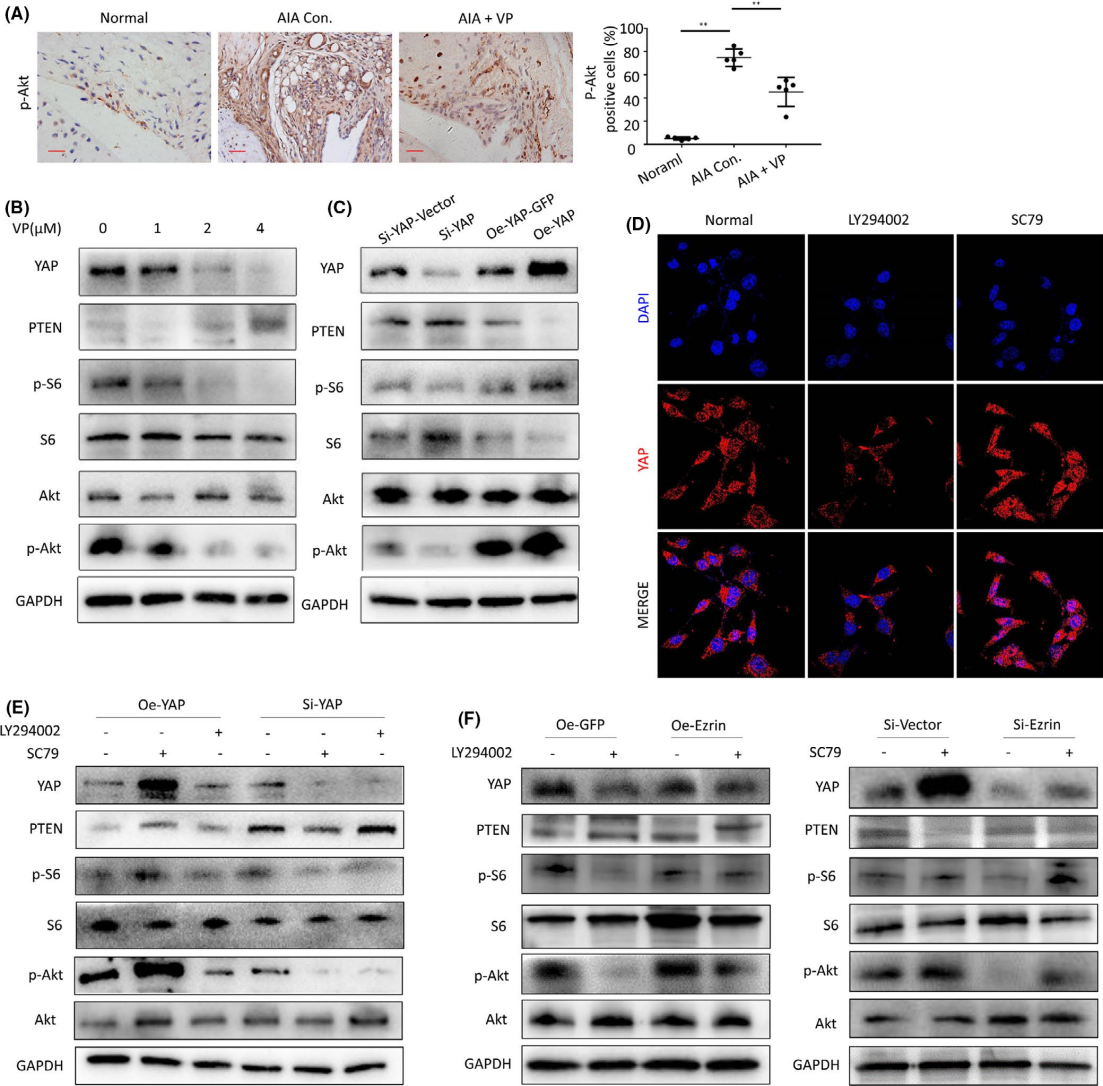
(A) p‐Akt protein levels in the IHC analysis and positive cells of the knee joints from treated AIA mice (Scale bar, 25 μm). (B) The Western blot results showed that the verteporfin treatment inhibited the protein expression of PI3K/Akt in HUVECs. (C) The Western blot was used to measure YAP protein in HUVECs following transfection and silencing of YAP and the protein expression of PI3K/Akt. (D) Representative images of YAP nuclear translocation affected by PI3K/Akt signalling pathway. (E) Western blot results showed that there was interaction between hippo‐YAP expression and PI3K/Akt signalling pathway. (F) Western blot results showed that there was interaction between Ezrin expression and PI3K/Akt signalling pathway

Prior research showed that the PI3K/Akt signalling pathway is a classical proliferation pathway and may be regulated by YAP. We found that the expression of p‐Akt was increased in different AIA groups, which suggested activation of the PI3K/Akt signalling pathway. However, the expression of p‐Akt in AIA mice treated with VP was found to be decreased compared to control mice (Figure 6A). We set out to validate these results and found that, when VP was added to HUVECs, YAP expression was inhibited with the increase in VP concentration, and the ratio of p‐S6/S6 and p‐Akt/Akt was decreased (Figure 6B). We next constructed an adenovirus to silence and overexpress YAP. Following transfection, we found that YAP silencing increased PTEN expression, although the expression of p‐S6 and p‐Akt and the ratio of p‐S6/S6 and p‐Akt/Akt were both decreased. The reverse was observed following YAP overexpression. Previous studies suggested interactions between the PI3K/Akt signalling pathway and the Hippo pathway. Indeed, we observed PI3K/Akt pathway activation during HUVEC cell proliferation (Figure 6C). We used Akt blockers or agonists to assess the effect of manipulation of the PI3K/Akt signalling pathway on YAP entry into the nucleus. When compared with the control group, the LY294002 (PI3K inhibitor) group resulted inhibition of the expression of YAP and impaired nuclear translocation of HUVECs; in contrast, the SC79 (Akt phosphorylation activator) group promoted expression and YAP nuclear translocation in HUVECs. At the same time, YAP expression and nuclear translocation were found to be inhibited in LY294002 group (Figure 6D). Our results confirmed interaction between the Hippo‐YAP and the PI3K / Akt signalling pathways (Figure 6E and 6F).

## 4 DISCUSSION

RA arthritis is a serious chronic autoimmune disease. In the early stages of RA, different degrees of microvascular formation occur in the synovial tissues, and a large number of immune‐inflammatory cells enter the joint tissues through the blood flow.^12^ Angiogenesis is a complex regulatory process of blood vessel formation that involves multiple factors. A large number of immune cells and inflammatory mediators promote the excessive proliferation of synovial cells in RA, and proliferation of local vascular skin cells gradually leads to the formation of pannus.^13^ At the same time, pannus formation also leads to the local proliferation of synovial tissues, which become even more invasive. With the aggravation of the disease course, pannus gradually erodes the articular cartilages and bones, and ultimately leads to joint damage.^14^, ^15^ In this study, we triggered arthritis in mice by using Freund's adjuvant, in order to simulate the pathogenesis of RA. Histology and immunostaining revealed excessive neovascularization in the synovial tissues of AIA mice. This phenomenon is reminiscent of the pathological RA process that occurs in humans.

Until now, the mechanisms underlying the angiogenesis process in RA are largely unknown. Increasing knowledge in this area may help determine more effective molecular targets of angiogenesis for the potential treatment of RA.

The membrane cytoskeleton junction protein (Ezrin) is one of the member of ERM (i.e. Ezrin, Radixin and Moesin) protein family. Previous studies have shown that Ezrin can participate in cell processes in health and disease relate to development, and metastasis of tumours, and cell adhesion, movement and angiogenesis through a variety of signalling pathways.^16^, ^17^, ^18^, ^19^ In this study, we observed that the proliferation activity of synovial vessels was enhanced and the Ezrin expression in the synovial vessels was also higher in specimens from patients with RA. We further explored the role of Ezrin protein in vascular endothelial cells and found it plays a role in the proliferation, migration and tube forming ability of vascular endothelial cells. Past studies have demonstrated that silencing Ezrin can increase the apoptosis rate of HUVECs and inhibit their proliferation. At the same time, Ezrin overexpression can affect the migration and angiogenesis of HUVEC cells by cytoskeleton remodelling. Our results are consistent with previous literature suggesting a role for Ezrin protein in the proliferation and migration of vascular endothelial cells.

Recent studies revealed that YAP plays several roles in regulating in angiogenesis. YAP is located on the chromosome 11q22.1, which encodes a proline‐rich phosphoprotein with a molecular weight of 65 kD that binds with the oncogene yes. YAP consists of one or two WW domains, 14–3–3 protein‐binding sites, PDZ binding motifs, amino‐terminal proline‐rich domains and coiled‐coil domains.^20^, ^21^ Because of its multiple domains, YAP can interact with a variety of proteins, regulate multiple cell signalling pathways and perform a variety of biological functions. Prior literature confirmed that YAP plays a role in regulating angiogenesis, which can promote endothelial cell proliferation and angiogenesis as well as induce vascular development.^22^ After entering the nucleus, the activated YAP binds to its ligand TEAD, affecting signal factors involved in cell migration and angiogenesis.^23^, ^24^ Under normal physiological circumstances, YAP is found to be phosphorylated, so the YAP‐TEAD pathway is not activated in normal tissues. Prior studies have also revealed abundant nuclear YAP expression in endothelial cells at the front of neovascularization, suggesting that a large amount of YAP is activated during angiogenesis, similar to that in other cell types and regulated via cell‐to‐cell contact.^25^ In addition, VE cadherin‐mediated PI3K/AKT signalling pathway regulates the YAP subcellular localization. Simultaneously, YAP in the nucleus can induce the expression of angiogenin‐2 (Ang‐2) and thereby promote angiogenesis germination and vascular remodelling.^26^ In addition, a number of studies have demonstrated that YAP promotes smooth muscle proliferation and migration through the inhibition of the expression of smooth muscle‐specific genes.^27^ In the process of smooth muscle development, YAP also promotes proliferation and inhibiting the differentiation of smooth muscle cells. YAP conditional knockout mice exhibit severe vascular abnormalities.^28^ However, in patients with RA, the mechanisms underlying YAP activation in the synovial vessels remains unclear.

Therefore, in this study, to set out to elucidate the crosstalk between YAP and Akt signalling and to clarify the significance of Ezrin as a protein critical to the regulatory machinery central to pathways involved in RA. We found that Ezrin regulated the activity of the Hippo‐YAP pathway. Silencing Ezrin downregulated the phosphorylation of YAP in the nucleus. The overexpression of Ezrin promoted the phosphorylation of YAP. Therefore, Ezrin seems to affect the nuclear translocation of YAP via regulation of YAP phosphorylation. To further confirm our results, we used VP, an inhibitor of YAP, mice experiments. Our results revealed that VP administration in AIA mice regulated the formation of synovial blood vessels and led to inhibition the process of synovitis in these animals. Furthermore, our results suggest that YAP plays a potential role in regulating the PI3K/Akt pathway. We constructed adenovirus to manipulate expression of YAP in HUVECs. Prior studies demonstrated that PI3K activity is regulated by the tumour suppressor PTEN.^29^, ^30^ Western blot results revealed decreased PTEN expression and activation of the PI3K/Akt signalling pathway in the YAP overexpression group. In contrast, in the YAP knockdown group, PTEN expression was increase and the PI3K/Akt signalling pathway was inhibited. Furthermore, we confirmed the effect of Ezrin manipulation on the PI3K/Akt pathway. The PI3K inhibitor, LY294002, was used to treat Ezrin overexpressed HUVECs, whereas the PI3K activator, SC79 was used to treat HUVECs in which Ezrin had been silenced. Our results showed significant elevation of the YAP, p‐Akt and p‐s6 proteins in Ezrin‐silenced HUVECs, whilst YAP, p‐Akt and p‐s6 proteins were significantly downregulated in Ezrin‐overexpressed HUVECs compared with their levels in the control group. These results confirmed that manipulation of the Ezrin pathway can affect the PI3K/Akt and YAP pathways. Our data further suggest that Ezrin plays an important role in maintaining synovial angiogenesis in RA through YAP and Akt signalling. Further studies on the Ezrin regulation of the angiogenesis are likely to reveal more additional functions for Ezrin and may provide further insight into the novel role of Ezrin in angiogenesis RA and may lead to the development of potential new therapeutic avenues for patients with RA.

Our study also has several limitations. The adjuvant‐induced arthritis models used in this study may not fully mimic the natural process of RA in patients. Therefore, our findings may not be generalizable to RA in humans.

## 5 CONCLUSION

**FIGURE 7:**
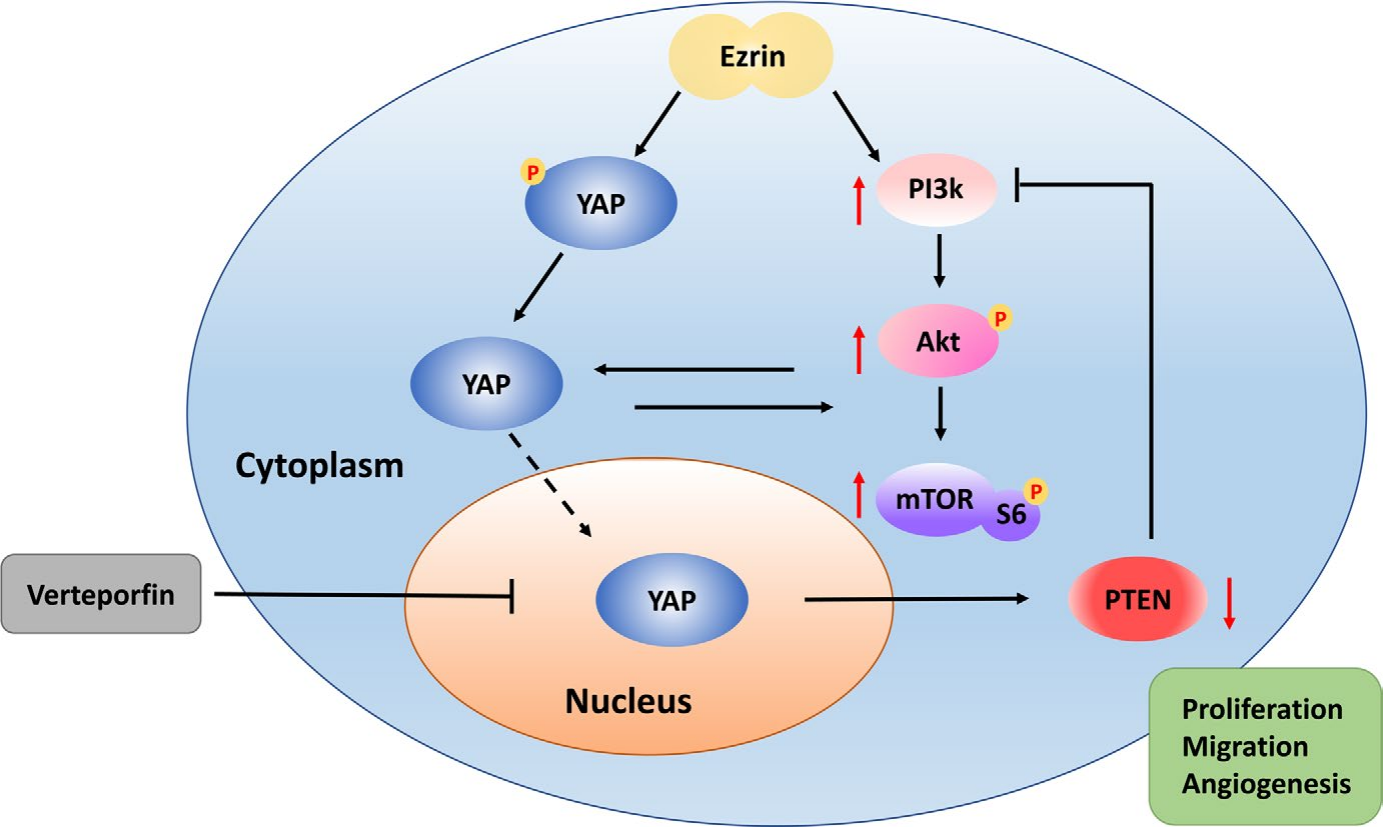
Schema of underlying mechanism of Ezrin induction of the synovial angiogenesis in rheumatoid arthritis

In conclusion, the results of the present study demonstrated that Ezrin expression was increased in RA synovial vessels. In addition, we also demonstrated that the PI3K/Akt pathway was activated by the Hippo‐YAP pathway. Ezrin was found to promote the activation and nuclear translocation of YAP, thereby playing a potential role in transcription. Our results suggest that activated YAP can further activate the PI3K/Akt pathway, which in turn promotes proliferation and vascular activities of vascular endothelial cells. Ezrin or Hippo‐YAP pathway inhibition was shown to inhibit the proliferation of RA synovial vessels as well as inhibit inflammatory processes of RA joints in mice (Figure 7). The results may provide a new direction for the treatment of RA synovial angiogenesis.

## CONFLICTS OF INTEREST

The authors declare no conflicts of interest.

## AUTHOR CONTRIBUTION

**Qiyue Chen:** Formal analysis (equal); Investigation (equal); Methodology (equal); Software (equal); Visualization (equal). **Kai Fan:** Formal analysis (equal); Investigation (equal); Validation (equal). **Xi Chen:** Validation (equal). **Xiaobo Xie:** Validation (equal). **Li Huang:** Validation (equal). **Guangbao Song:** Conceptualization (equal); Project administration (lead); Resources (equal). **Weizhong Qi:** Conceptualization (equal); Data curation (equal); Resources (equal); Supervision (equal); Writing‐original draft (lead); Writing‐review & editing (lead).

## Data Availability

The data presented in this study are available on request from the corresponding author.
