# Correction to Ezrin regulates synovial angiogenesis in rheumatoid arthritis through YAP and Akt signalling

**DOI:** 10.1111/jcmm.17987

**Published:** 2023-11-27

In Qiyue Chen et al.,^1^ there was an error during the preparation of the P‐AKT immunohistochemistry image for the normal control group in Figure 6A. The corrected figure is provided below. The authors affirm that all the results and conclusions of this article remain unchanged.

**FIGURE 6:**
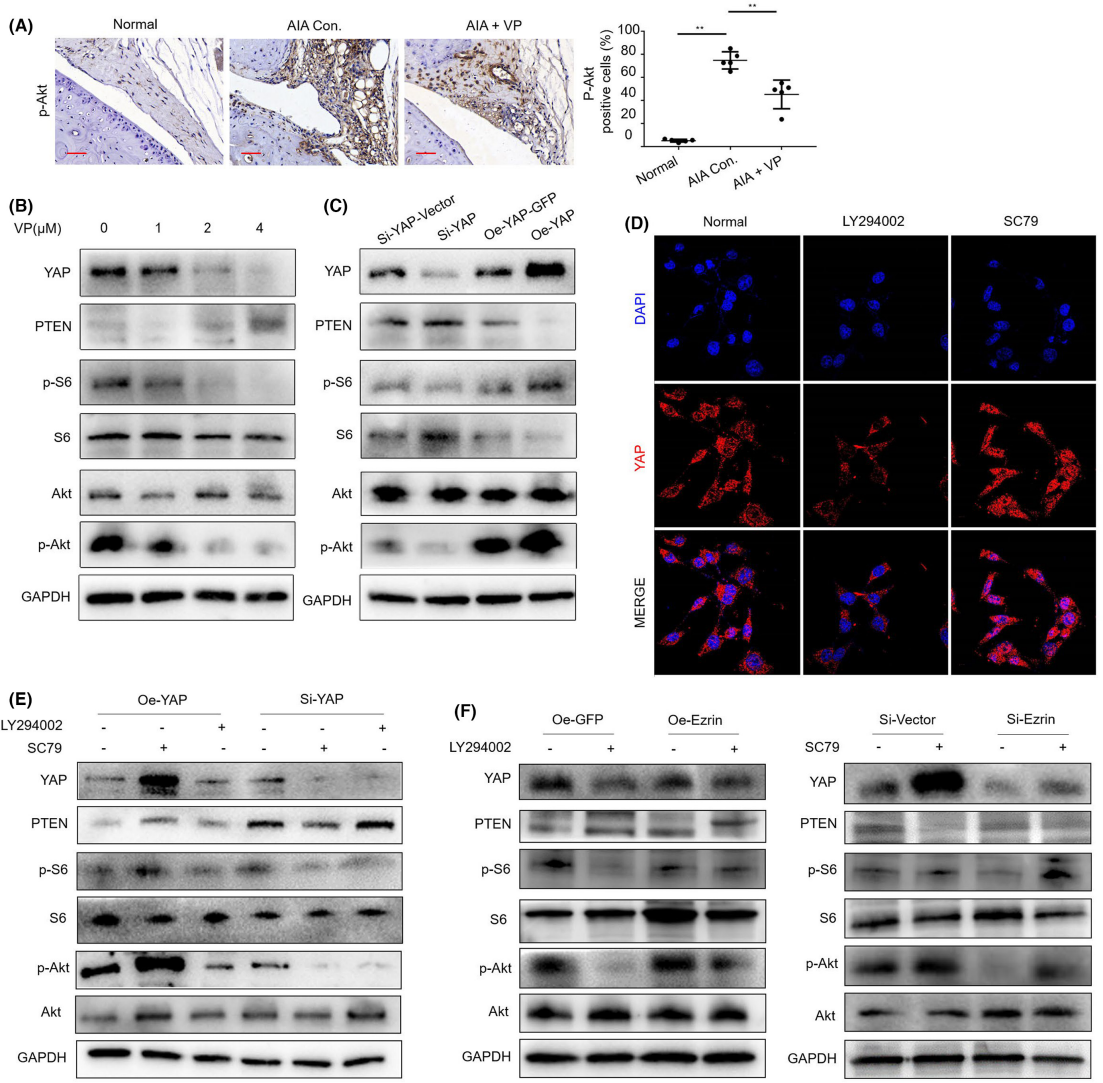
(A) p‐Akt protein levels in the IHC analysis and positive cells of the knee joints from treated AIA mice (Scale bar, 50 μm). (B) The western blot results showed that the verteporfin treatment inhibited the protein expression of PI3K/Akt in HUVECs. (C) The western blot was used to measure YAP protein in HUVECs following transfection and silencing of YAP and the protein expression of PI3K/Akt. (D) Representative images of YAP nuclear translocation affected by PI3K/Akt signalling pathway. (E) Western blot results showed that there was an interaction between hippo‐YAP expression and PI3K/Akt signalling pathway. (F) Western blot results showed that there was an interaction between Ezrin expression and PI3K/Akt signalling pathway.
